# Health Status and Risk Factors among Adolescent Survivors One Month after the 2014 Ludian Earthquake

**DOI:** 10.3390/ijerph120606367

**Published:** 2015-06-04

**Authors:** Bihan Tang, Yang Ge, Chen Xue, Peng Kang, Yuan Liu, Xu Liu, Zhipeng Liu, Wenya Yu, Lulu Zhang

**Affiliations:** Institute of Military Health Management, Second Military Medical University, 800 Xiangyin Rd, Shanghai 200433, China; E-Mails: mangotangbihan@126.com (B.T.); geyang_happy@163.com (Y.G.); xue1990chen@163.com (X.C.); kangpengpkpk@sina.com (P.K.); yawnlau@126.com (Y.L.); xuliuaqua@126.com (X.L.); liuzhipeng__1987@sina.com (Z.L.); jsjyyuwenya@sina.cn (W.Y.)

**Keywords:** adolescents, health status, earthquake, risk factor

## Abstract

Background: An earthquake struck Ludian in Yunnan Province (China) on 3 August 2014, resulting in 3143 injuries, 617 deaths, and 112 missing persons. Our study aimed at estimating the health status and associated determinants among adolescent survivors after the Ludian earthquake. *Methods***:** A cross-sectional survey of 845 was conducted at the Ludian No. 1 Middle School. Descriptive statistics, t-tests, ANOVA and stepwise linear regression analysis were used for data analysis. *Results***:** The mean scores on the physical component summary (PCS) and mental component summary (MCS) were 46.23 (SD = 7.10) and 36.34 (SD = 7.09), respectively. Lower PCS scores in the aftermath of an earthquake were associated with being trapped or in danger, being female, being an ethnic minority, injury to self and house damage, while lower MSC scores were associated with fear during the earthquake, Han ethnicity, death in the family, not being involved in the rescue and low household income. *Conclusions*: In our study, significant associations between demographic, socio-economic, and trauma-related experiences variables and overall physical and mental health of adolescent survivors were presented. The results of this study help expand our knowledge of health status among adolescent survivors after the Ludian earthquake.

## 1. Introduction

An earthquake measuring 6.5 on the magnitude scale and 8.0 on the intensity scale, struck Ludian in Yunnan Province (China) at 4:30 p.m. local time on Sunday, 3 August 2014. Often referred to as the Ludian earthquake, this disaster resulted in 3143 injuries, 617 deaths, and 112 missing persons. While an earthquake may cause disastrous loss of life, homes, and livelihoods instantly, it can also cause serious psychological effects for survivors as well as physical injuries and the exacerbation of existing physical conditions [1,2]. Mental and physical impairment following an earthquake will inevitably affect one’s health status. The instruments of health status, such as the 36-item short form health survey (SF-36), and more recently the acute version of the Short Form-12 (SF-12), had brought significant benefit for all researches concerned with health outcomes [3].

There is an emerging literature on health status among survivors of earthquakes. Many studies have emphasized that earthquakes impair the health status of survivors [1,2,4,5,6,7,8], and several studies have been conducted to explore the determinants of poor health outcomes [9,10,11,12,13]. However, most of these studies focused on the health conditions of adults. Only three focused on the health status of children and adolescents and the sample numbers were limited (ranging from 511 to 596) [8,9,13]. Enabling children and adolescents who have survived disasters to achieve and maintain the same standards of health as other children and adolescents has been a goal of international communities [14].

In our study, a population-based survey of 845 adolescents aged between 14 and 19 years was conducted in Ludian County one month after the disaster, to estimate the health status and associated determinants, and provide evidence-based suggestions for specific health interventions in vulnerable populations.

## 2. Method

### 2.1. Study Design and Participants

A cross-sectional survey was conducted in September 2014, by which time one month had passed since the event. Our study participants comprised all senior students (Grade 1–Grade 3) who were present at the Ludian No. 1 Middle School at the time of our evaluation and had experienced the Ludian earthquake themselves. A total of 1030 senior students were approached: 113 were excluded from our investigation since they did not experience the Ludian earthquake themselves and 11 refused to participate in the investigation. Thus, 906 senior students completed the questionnaire, and 61 questionnaires were excluded because of missing items. The total number of completed questionnaires was 845.

### 2.2. Data Collection

The survey took place in the classrooms of the school. Two survey teams were established, each comprised of three graduate students and one professional staff member of the Second Military Medical University. Professional staff members briefed prospective respondents about the details of the study and answered questions related to the study since the comprehension ability of the middle school students was limited. Approval was obtained from the school principal who made an announcement about the study to the students. The study obtained the approval from the ethics committee of Second Military Medical University, and all the participants were voluntary to participate in our survey (Project identification code: 91224005).

### 2.3. Measurements

The social and demographic information included gender, age, ethnicity, annual household income, and disease history. Earthquake-related experiences were assessed with an inventory adapted from a commonly used earthquake exposure scale [15]. “Disease history” did not refer to any injuries caused by the earthquake. It included both physical and mental illness that can influence one’s daily life, such as autism, bone fractures, poliomyelitis, congenital heart disease and so on. “Injury” referred to all injuries caused by the Ludian earthquake, regardless of whether the injuries had been healed during our investigation. 

The health status of the students was measured using the acute version of the Short Form-12 (SF-12). The SF-12 is a subset of the 36-item short form health survey (SF-36) scale. Translated into multiple languages, the SF-12 includes one item from each of the bodily pain, general health, vitality, and social functioning scales and two items from each of the physical functioning, role-physical, role-emotional, and mental health scales of the SF-36 [16]. In addition, the SF-12 assesses overall physical and mental functioning using two summary scales: physical component summary (PCS) and mental component summary (MCS) [17]. The participants were asked to report the general health status by recalling their personal experience in the previous four weeks and responding to the 12 items. It takes only two to three minutes to complete the 12 items. Higher summary PCS and MCS scores are indicative of better health [18]. The Chinese version of the SF-12 used in this study (Appendix) has been validated in Chinese adolescents [19].

### 2.4. Data Analysis

All analyses were performed using the Statistical Package for the Social Sciences (SPSS) Version 11.0 (SPSS Inc., Chicago, IL, USA). We first calculated the descriptive statistics (frequencies, percentages, means, and standard deviations [SD]); then, t-tests (for two-group comparisons) and analysis of variance (ANOVA, for multi-group comparisons) were used to evaluate differences in continuous variables if the data were in accordance with assumptions regarding the normal distribution and homogeneity of variance; otherwise, a non-parametric method was used. Stepwise linear regression analysis was performed to identify predictors of PCS and MCS, and beta coefficients (B), standardized error of the coefficient (S.E.B), and standardized regression coefficient (Beta) were reported. The criterion for statistical significance was *p* = 0.05.

## 3. Results

### 3.1. Social-Demographic Characteristics and Earthquake-Related Experiences

Table 1 summarizes the social-demographic characteristics and earthquake-related experiences of the participants. The mean scores on the PCS and MCS were 46.23 (SD = 7.10) and 36.34 (SD = 7.09), respectively. Of the 845 participants, 456 (53.96%) were male, 803 (95.03%) were of Han descent (the major ethnic population in China), and over half (58.7%) were 16 or 17 years old. Most families (94.67%) earned less than 8000 Yuan per year, and 688 participants (81.42%) had no history of disease. With regard to earthquake-related experiences, only 83 participants (9.82%) were injured due to the earthquake, 272 (32.19%) reported that a family member was injured in the earthquake, 189 (22.39%) reported that a family member died in the earthquake, most (816; 96.57%) felt fear during the earthquake, and 317 (37.51%) were trapped or in danger in the earthquake. Few houses (15; 1.78%) were in excellent condition without damage. After the earthquake, 333 participants (39.41%) were involved in the rescue. Over half of participants (485; 57.4%) had experienced an earthquake before, and most participants (740; 87.57%) had received escape training before the earthquake.

**Table 1:** Demographics and earthquake-related experiences among adolescent survivors of the Ludian earthquake, China.

Variable	N	Percentage (%)	PCS		MSC	
			Mean	*p*	Mean	*p*
Total	845	–	46.23 (7.10)	−	36.34 (7.09)	−
Gender				0.002		0.844
Male	456	54.0	46.93 (7.17)		36.29 (7.03)	
Female	389	46.0	45.40 (6.93)		36.39 (7.17)	
Age				0.879		0.502
14	28	3.3	46.12 (6.26)		38.07 (6.16)	
15	145	17.2	46.21 (6.47)		36.56 (6.57)	
16	268	31.7	45.98 (7.52)		36.25 (7.20)	
17	228	27.0	46.67 (6.81)		36.01 (7.24)	
18	119	14.1	46.31 (7.46)		35.92 (7.02)	
19	57	6.8	45.53 (7.47)		37.52 (7.76)	
Ethnicity				0.002		0.005
Han	803	95.0	46.40 (7.05)		36.18 (7.03)	
Minority	42	5.0	42.98 (7.33)		39.35 (7.66)	
Annual household income (yuan)				0.278		0.005
1000–4000	609	72.1	46.41 (7.10)		35.86 (6.97)	
4000–8000	191	22.6	45.48 (7.12)		37.94 (7.31)	
8000–12000	38	4.5	47.33 (6.87)		35.83 (6.93)	
>12000	7	0.8	44.45 (6.67)		36.81 (6.96)	
History of disease				0.072		0.267
No	688	81.4	46.44 (7.03)		36.21 (6.96)	
Yes	157	18.6	45.31 (7.32)		36.91 (7.63)	
Injury to self due to earthquake				0.004		0.407
No	762	90.2	46.46 (7.03)		36.27 (7.09)	
Yes	83	9.8	44.08 (7.40)		36.95 (7.08)	
Injury in the family due to earthquake				0.027		0.137
No	573	67.8	46.60 (7.08)		36.09 (7.07)	
Yes	272	32.2	45.44 (7.09)		36.87 (7.10)	
Death in the family due to earthquake				0.224		0.008
No	655	77.6	46.06 (7.31)		36.69 (6.79)	
Yes	189	22.4	46.77 (6.30)		35.15 (7.94)	
Fear during the earthquake				0.489		0.029
No	29	3.4	45.09 (6.59)		38.95 (7.94)	
A little	242	28.6	46.33 (6.92)		37.03 (6.72)	
Some	176	20.8	46.51 (6.96)		36.48 (7.57)	
Much	253	29.9	46.52 (7.15)		35.90 (7.06)	
Extremely	145	17.2	45.41 (7.55)		35.25 (6.78)	
Trapped or in danger				<0.001		0.001
No	528	62.5	47.06 (6.99)		35.99 (6.62)	
Yes	317	37.5	44.83 (7.07)		36.92 (7.78)	
House damage due to earthquake				0.020		0.091
No	15	1.8	45.68 (8.39)		40.26 (6.24)	
Slight damage	142	16.8	47.61 (6.39)		36.07 (6.88)	
Moderate damage	264	31.2	46.69 (6.94)		35.76 (6.82)	
Partial collapse	256	30.3	45.69 (7.73)		36.42 (7.37)	
Complete collapse	168	19.9	45.18 (6.60)		37.00 (7.22)	
Involved in the rescue				0.159		0.228
No	512	60.6	46.46 (7.29)		35.83 (6.89)	
Yes	333	39.4	45.86 (6.79)		37.13 (7.32)	
Psychological counseling				0.302		0.561
No	481	56.9	46.44 (6.66)		36.22 (6.92)	
Yes	364	43.1	45.94 (7.63)		36.50 (7.30)	
Experienced previous earthquake				0.130		0.374
No	360	42.6	46.65 (7.43)		36.09 (7.10)	
Yes	485	57.4	45.91 (6.82)		36.53 (7.08)	
Escape training				0.321		0.238
No	105	12.4	45.58 (6.75)		37.10 (7.84)	
Yes	740	87.6	46.32 (7.14)		36.23 (6.97)	

### 3.2. Factors Associated with Physical Component Summary 

Bivariate analyses (Table 1) were conducted to assess the health status during the aftermath of the earthquake, as measured by the PCS. Significant PCS scores were associated with gender (*p*= 0.002), ethnicity (*p* = 0.002), injury to self (*p* = 0.004), injury in the family (*p* = 0.027), being trapped or in danger (*p* < 0.001), and house damage (*p* = 0.02). Multiple stepwise regression analyses (Table 2) revealed that lower PCS scores in the aftermath of an earthquake were associated with being trapped or in danger (*p* = 0.001), being female (*p* = 0.003), being an ethnic minority (*p* = 0.007), injury to self (*p* = 0.019), and house damage (*p* = 0.032).

**Table 2:** Multiple stepwise regression analysis of factors independently associated with PCS and MCS.

Label	B ^a^	S.E.B ^b^	B ^c^	*p* ^d^
**Dependent variable: PCS**				
Trapped or in danger (No (0), Yes (1))	−1.676	0.505	−0.114	0.001
Gender (Male (0),Female (1))	−1.428	0.480	−0.100	0.003
Ethnicity (Han (0),Minority (1))	−2.970	1.105	−0.091	0.007
Injury to self due to earthquake (No (0), Yes (1))	−1.898	0.811	−0.080	0.019
House damage due to earthquake (No (0), Yes (1))	−0.499	0.232	−0.073	0.032
**Dependent variable: MCS**				
Fear during the earthquake (No (0), Yes (1))	−0.591	0.208	−0.096	0.000
Ethnicity (Han(0),Minority(1))	3.120	1.103	0.096	0.005
Death in the family due to earthquake (No (0), Yes (1))	−1.605	0.576	−0.094	0.005
Involved in the rescue (No (0), Yes (1))	1.230	0.491	0.085	0.005
Annual household income (1000–4000 (0), 4000–8000 (1), 8000–12000 (2), >12000 (3))	0.968	0.398	0.082	0.012

**^a^** Unstandardized regression coefficient; **^b^** Standardized error of coefficient; **^c^** Standardized regression coefficient; **^d^** The inclusion criteria of the stepwise regression was *p* = 0.05; exclusion criteria was *p* = 0.10.

### 3.3. Factors associated with Mental Component Summary

Bivariate analyses (Table 1) were conducted to assess the health status during the aftermath of the earthquake, as measured by the MCS. Significant MCS scores were associated with ethnicity (*p* = 0.005), annual household income (*p* = 0.005), death in the family (*p* = 0.008), fear during the earthquake (*p* = 0.029), and being trapped or in danger (*p* = 0.001). Multiple stepwise regression analyses (Table 2) revealed that lower MSC scores in the aftermath of the earthquake were associated with fear during the earthquake (*p* < 0.001), Han ethnicity (*p* = 0.005), death in the family (*p* = 0.005), not being involved in the rescue (*p* = 0.005), and low household income (*p* = 0.012).

## 4. Discussion

Experiencing a disastrous earthquakeresults in mental and physical impairment among adolescent survivors. Using the SF-12 as an instrument to assess the impact of the Ludian earthquake on the health status of adolescent survivors, we found that the Ludian earthquake survivors had both worse PCS (46.2 *vs.* 49.8) and MCS (36.3 *vs.* 45.4) than the general adolescent population in China [19]. This result indicated that the health outcomes of adolescent survivors had been impaired by the Ludian earthquake. However, there were no values for the same adolescent population before the earthquake in our study. As a result, we cannot figure out what the acute extent the earthquake influenced the adolescents’ health status there.

With respect to risk factors related to physical health status among adolescent survivors, we found that the following variables had a strong negative association with physical health: female gender, being trapped, injury, minority ethnicity, and house damage. Research shows that girls are more worried about their bodies than boys are; they are more concerned with themselves and their well-being, and are more sensitive [20]. Moreover, adolescents experience increasing discrepancies in their physical and hormonal development as they age. Menstruation disorders have become the most common health problems following colds and influenza [21]. The hormonal fluctuations experienced by female adolescents may also lead to poorer perceptions of their own health status. Those who were trapped during the earthquake or experienced house damage often experienced physical injuries. In addition, survivors who experienced severe house damage had to live in temporary shelters with poor conditions, high population density, and poor ventilation. The risk of occurrence and spread of acute respiratory infections among the victims that lived in the temporary settlements was very high, particularly for acute infections such as the common cold [5]. Published studies have also found an association between physical health and housing conditions [12,22,23].

Our study also indicated an interesting phenomenon, that is, people of ethnic minorities tended to experience worse physical health status but better mental health status compared to those of Han nationality. However, we only included 42 participants (4.97%) from ethnic minorities; further research is needed to investigate differences in psychological and biological mechanisms between ethnic minorities and the Han population in the face of a traumatic disaster.

With regard to psychological health status, we also found that being scared and losing family members during the earthquake corresponded to lower MCS scores among the adolescent survivors. One study found that adolescents who had lost family members were 6.6 and 4.1 times more likely to develop PTSD and depression than those who did not, respectively [9]. Another study also indicated that adolescents who experienced the death of family members were more likely to have symptoms of anxiety [24]. It is likely that fear *per se* does not decrease one’s mental health status; this effect was perhaps mediated instead by the subjective experience of the natural disaster and personality type [25]. For example, individuals with high neuroticism tend to be more reactive and sensitive to adverse events, possibly increasing their risk of developing depression [26]. We also found that participating in rescue work after the earthquake and high family income corresponded to higher MCS scores. Adolescents who are involved in rescue work right after a disaster show better resilience to the adversity than those who refuse to participate in rescue work [27]. In addition, teachers and parents praised adolescents who were involved in rescue work and talked about their helpful deed to others. In this way, these adolescents’ felt a great deal of self-respect and self–satisfaction, which most likely helped increase their mental health status. In terms of family income, we expect that level of household income indirectly influenced economic resources, social status, social networks, and health behavior. Thus, those with a higher family income might use better coping methods because of their parents’ greater social and economic resources, ultimately contributing to better mental health status after facing natural disasters. However, since the adolescents did not contribute to the family income, information about household income obtained from them may not always have been accurate. Further research is needed to examine the effects of family income on mental health status in the future.

The current study has a few limitations that are worthy of note. First, because it was an exploratory cross-sectional study, it was difficult to conclude to what extent the earthquake had an effect on those who experienced it, as no pre-disaster comparison data was available. Second, although the study was conducted adequately by trained investigators, data collection relied solely on retrospective self-reports of the survivors, which can be subject to recall and desirability biases. Third, the study sample was recruited from one of the most affected middle schools in Ludian County, meaning that it is not appropriate to generalize these conclusions to those of less affected regions. Despite these limitations, the present study is one of only a few cross-sectional studies focusing on health status and its risk factors in adolescent populations following an earthquake. In our study, significant associations between demographic, socio-economic, and trauma-related experiences variables and overall physical and mental health of adolescent survivors were presented. 

## 5. Conclusions

In conclusion, the results of this study help expand our knowledge of health status of adolescent survivors after the Ludian earthquake, lead to greater vigilance of and attention to those adolescence who were most compromised by the earthquake. This study provides important information for monitoring the course of recovery of adolescents after earthquake and is of great significance for public awareness and sensitive care for those who were the most affected. Specific care and psychological health interventions should be provided to those adolescents whose health status was relatively low.

## Appendix

The Chinese version of the SF-12 used in our study.

**Table:**

1. In general, would you say your health is excellent, very good, good, fair, or poor?
2. Limitations in moderate activities, such as moving a table, or doing some cleaning?
3. Limitations in climbing several flights of stairs?
4. During the past 4 weeks, have you accomplished less than you would like as a result of your physical health?
5. During the past 4 weeks, were you limited in the kind of study or other regular daily activities you do as a result of your physical health?
6. During the past 4 weeks, have you accomplished less than you would like as a result of any emotional problems, such as feeling depressed or anxious?
7. During the past 4 weeks, did you not do study or other regular activities as carefully as usual as a result of any emotional problems, such as feeling depressed or anxious?
8. During the past 4 weeks, how much did pain interfere with your normal study?
9. How much of the time during the past 4 weeks have you felt calm and peaceful?
10. How much of the time during the past 4 weeks did you have a lot of energy?
11. How much of the time during the past 4 weeks have you felt downhearted and blue?
12. How much of the time have your physical health or emotional problems interfered with your social activities, like visiting with friends or relatives?
